# Diffusion MRI microstructure models with in vivo human brain Connectome data: results from a multi‐group comparison

**DOI:** 10.1002/nbm.3734

**Published:** 2017-06-23

**Authors:** Uran Ferizi, Benoit Scherrer, Torben Schneider, Mohammad Alipoor, Odin Eufracio, Rutger H.J. Fick, Rachid Deriche, Markus Nilsson, Ana K. Loya‐Olivas, Mariano Rivera, Dirk H.J. Poot, Alonso Ramirez‐Manzanares, Jose L. Marroquin, Ariel Rokem, Christian Pötter, Robert F. Dougherty, Ken Sakaie, Claudia Wheeler‐Kingshott, Simon K. Warfield, Thomas Witzel, Lawrence L. Wald, José G. Raya, Daniel C. Alexander

**Affiliations:** ^1^ Centre for Medical Image Computing Department of Computer Science, University College London UK; ^2^ Department of Radiology New York University School of Medicine USA; ^3^ Department of Neuroinflammation, Institute of Neurology University College London UK; ^4^ Computational Radiology Laboratory, Boston Children's Hosp. Harvard University USA; ^5^ Philips Healthcare Guildford Surrey UK; ^6^ Chalmers University of Technology Gothenburg Sweden; ^7^ Centro de Investigacion en Matematicas AC Guanajuato Mexico; ^8^ Athena Project‐Team INRIA Sophia Antipolis ‐ Méditerranée France; ^9^ Lund University Bioimaging Center Lund University Sweden; ^10^ Erasmus Medical Center and Delft University of Technology the Netherlands; ^11^ eScience Institute University of Washington USA; ^12^ Center for Cognitive and Neurobiological Imaging Stanford University USA; ^13^ Imaging Institute The Cleveland Clinic Cleveland USA; ^14^ A.A. Martinos Center for Biomedical Imaging, MGH Harvard University USA

**Keywords:** brain microstructure, Connectome, diffusion MRI, fornix, genu, model selection

## Abstract

A large number of mathematical models have been proposed to describe the measured signal in diffusion‐weighted (DW) magnetic resonance imaging (MRI). However, model comparison to date focuses only on specific subclasses, e.g. compartment models or signal models, and little or no information is available in the literature on how performance varies among the different types of models. To address this deficiency, we organized the ‘White Matter Modeling Challenge’ during the International Symposium on Biomedical Imaging (ISBI) 2015 conference. This competition aimed to compare a range of different kinds of models in their ability to explain a large range of measurable in vivo DW human brain data. Specifically, we assessed the ability of models to predict the DW signal accurately for new diffusion gradients and b values. We did not evaluate the accuracy of estimated model parameters, as a ground truth is hard to obtain. We used the Connectome scanner at the Massachusetts General Hospital, using gradient strengths of up to 300 mT/m and a broad set of diffusion times. We focused on assessing the DW signal prediction in two regions: the genu in the corpus callosum, where the fibres are relatively straight and parallel, and the fornix, where the configuration of fibres is more complex. The challenge participants had access to three‐quarters of the dataset and their models were ranked on their ability to predict the remaining unseen quarter of the data. The challenge provided a unique opportunity for a quantitative comparison of diverse methods from multiple groups worldwide. The comparison of the challenge entries reveals interesting trends that could potentially influence the next generation of diffusion‐based quantitative MRI techniques. The first is that signal models do not necessarily outperform tissue models; in fact, of those tested, tissue models rank highest on average. The second is that assuming a non‐Gaussian (rather than purely Gaussian) noise model provides little improvement in prediction of unseen data, although it is possible that this may still have a beneficial effect on estimated parameter values. The third is that preprocessing the training data, here by omitting signal outliers, and using signal‐predicting strategies, such as bootstrapping or cross‐validation, could benefit the model fitting. The analysis in this study provides a benchmark for other models and the data remain available to build up a more complete comparison in the future.

**Abbreviations used**
CT: computerized tomography
CV: cross‐validation
DBF: diffusion basis function
DF: diffusion function
DT: diffusion tensor
DTI: diffusion tensor imaging
DW: diffusion‐weighted
EN: elastic net
ISBI: International Symposium on Biomedical Imaging
LASADD: Linear Acceleration of Sparse and Adaptive Diffusion Dictionary
LS: least‐squares
MRI: magnetic resonance imaging
PDD: principal diffusion directions
RSI: restriction spectrum imaging
ROI: region of interest
SFM: sparse fascicle model
SNR: signal‐to‐noise ratio
SSE: sum of squared errors
TE: echo time
WM: white matter

## 1 INTRODUCTION

Diffusion‐weighted (DW) magnetic resonance imaging (MRI) can provide unique insights into the microstructure of living tissue and is increasingly used to study the microanatomy and development of normal functioning tissue as well as its pathology. Mathematical models for analysis and interpretation have been crucial for the development and translation of DW‐MRI. Even though diffusion tensor imaging (DTI),^1^ which is based on a simple Gaussian model of the DW‐MRI signal, has shown promise in clinical applications,^2^ e.g. Alzheimer's disease,^3^ multiple sclerosis^4^ or brain tumors,^5^ a much wider variety of DW‐MRI models has been proposed to extract more information from the DW signal.

Models generally fall between two extremes: ‘models of the tissue’ and ‘models of the signal’. Models of the tissue^6^, ^7^, ^8^, ^9^, ^10^, ^11^, ^12^, ^13^, ^14^, ^15^, ^16^, ^17^ describe the underlying tissue microstructure in each voxel explicitly with a multi‐compartment approach.^18^, ^19^, ^20^ Models of the signal focus on describing the DW signal attenuation without describing the underlying tissue composition that gives rise to the signal explicitly.^21^, ^22^, ^23^, ^24^, ^25^, ^26^, ^27^, ^28^, ^29^ Other approaches fall between these two classes and include some features of the tissue, such as the distribution of fibre orientations, but often describe the signal from individual fibres without modelling the fibre composition explicitly.^30^, ^31^, ^32^, ^33^, ^34^, ^35^, ^36^, ^37^, ^38^, ^39^, ^40^

Despite this explosion of DW‐MRI models, a broad comparison on a common dataset and within a common evaluation framework is lacking, so little is understood about which models are more plausible representations or explanations of the signal. Panagiotaki et al.^18^ established a taxonomy of diffusion compartment models and compared 47 of them using data from the fixed corpus callosum of a rat acquired on a pre‐clinical system. Later, Ferizi et al.^39^ performed a similar experiment using data from a live human subject, while Ferizi et al.^41^, ^42^ explored a different class of models, which aim to capture fibre dispersion. Rokem et al.^43^ compared two classes of models using cross‐validation and test–retest accuracy. All these studies^18^, ^43^, ^44^ aim to evaluate variations with specific classes of models with all other variables of the parameter estimation pipeline (i.e. noise model, fitting routine, etc.) fixed. While this provides fundamental insight into which compartments are important in compartment models, questions remain about the broader landscape of models; in particular, which classes of models explain the signal best and how strongly performance depends on the choice of parameter‐estimation procedure.

Publicly organized challenges provide a unique opportunity to bring a research community together to gain a quantitative and unbiased comparison of a diverse set of methods applicable to a particular data‐processing task. Such publicly organized challenges have helped to establish a common ground for the evaluation of competing methods in a variety of imaging‐related tasks, e.g. in brain MR image registration^45^ and segmentation.^46^ In DW‐MRI, public challenges have focused on recovering synthetic intra‐voxel fibre configurations^47^ or evaluating tractography techniques^48^, ^50^ and have been very successful at driving research and translation forward. Another interesting comparison of reconstruction methods using DW‐MRI data was based on the signal acquired from a physical phantom.^49^ Here we report on such a community‐wide challenge to model the variation of DW‐MRI signals at the voxel level in the in vivo human brain.

Modelling the diffusion signal is a key step in realizing practical and reliable quantitative imaging techniques based on diffusion MRI. The challenge in the area is to extract the salient features from the diffusion signal and relate them to the principal features of the underlying tissue (e.g. in the case of brain white matter (WM) the fibre orientation, axonal packing and axonal size). Three distinct questions arise.

- Given the richest possible dataset that samples the space of achievable measurements as widely as possible, which mathematical model can capture best the intrinsic variation of the acquired signal?
- Which tissue features can be derived from the model?
- What subset of those features can we estimate from limited acquisition time on a standard clinical scanner and what dataset best supports such estimates?

The intuition gained from (i) is generalizable over a wide range of applications, while (ii) and (iii) are highly dependent on the MRI study design and the available hardware. Therefore, our challenge focuses on question (i), as an understanding of (i) is necessary to inform (ii) and (iii). To that end, we acquire the richest possible dataset using the most powerful hardware available and the most motivated subject available (UF). Specifically, we use the Connectome scanner,^51^ which is unique among human scanners in having 300 mT/m gradients, rather than 40 mT/m as is typical of state‐of‐the‐art human scanners. Preclinical work by Dyrby et al.^13^ highlights the benefits of such strong gradients and the first results from the Connectome scanner^42^, ^52^, ^53^, ^54^ are now starting to verify those findings.

This kind of model comparison, based on prediction error, is a common and crucial part of the development of any statistical model‐based estimation applications. Burnham and Anderson^55^ explain how and why such comparisons should be performed to reject models that are theoretically plausible but not supported by the data. To that end, we used a uniquely rich dataset acquired on the Connectome system^42^ composed of around 5000 points in *q* space with, for each shell, a unique combination of gradient strength, diffusion time, pulse width and echo time. This offers the opportunity for the comparison of the many different types of models within a common framework, over a very wide range of measurement space. Using this rich dataset, we organized the White Matter Modeling challenge, held during the 2015 International Symposium on Biomedical Imaging (ISBI) in New York. The goal of the challenge was to evaluate and compare the models in two different tissue configurations that are common in the brain: (1) a WM region of interest where fibres are relatively straight and parallel, specifically the genu of the corpus callosum; and (2) a region in which the fibre configuration is more complex, specifically the fornix. Challenge participants had access to three‐quarters of each whole dataset; the participating models were evaluated on how well they predicted the remaining ‘unseen’ part of the data. As announced before the challenge, the final ranking was based exclusively on the performance on the genu data. In this article, however, we include results from both the genu and the fornix.

The article is organized as follows. We first describe in section 2 the experimental protocol, data post‐processing and preparation of the training and testing data for the challenge. We then present the methods for ranking the models and tabulate the various models involved in the competition succinctly. We report the challenge results in section 3 and discuss these results in section 4; a more detailed description of the models follows in the Appendix.

## 2 MATERIAL AND METHODS

### 2.1 The complete experiment protocol

One healthy volunteer was scanned over two non‐stop 4 h sessions. The imaged volume comprised twenty 4 mm thick whole‐brain sagittal slices covering the corpus callosum left–right. The image size was 110×110 and the in‐plane resolution 2×2 mm^2^. 45 unique and evenly distributed diffusion directions (taken from http://www.camino.org.uk) were acquired for each shell, with both positive and negative polarities; these directions were the same in each shell. We also included 10 interleaved *b*=0 measurements, leading to a total of 100 measurements per shell. Each shell had a unique combination of Δ={22,40,60,80,100,120} ms, *δ*={3,8} ms and |**G**|={60,100,200,300} mT/m (see Table 1). The measurements were randomized within each shell, whereas the gradient strengths were interleaved. We inspected the images visually and did not observe any obvious shifts from gradient heating. The minimum possible echo time (TE) for each gradient duration and diffusion time combination was chosen to enhance signal‐to‐noise ratio (SNR) and potential estimation of compartment‐specific relaxation constants. The SNR of *b*=0 images was 35 at TE = 49 ms and 6 at TE = 152 ms. The SNR was computed by assessing the signal mean and noise variance across the selected WM voxels on multiple *b*=0 images. In both cases these estimates matched reasonably well. More details about the acquisition protocol can be found in Ferizi et al.^42^

**Table 1:** The scanning protocol used, acquired in ∼8 hours over two non‐stop sessions. The protocol has 48 shells, each with 45 unique gradient directions (‘blip‐up‐blip‐down’)

**Acquisition Protocol**									
	***δ***=**3** ***m*** ***s***					***δ***=**8** ***m*** ***s***			
	**Δ**	**TE**	|**G**|	**b**		**Δ**	**TE**	|**G**|	**b**
**Nr**	**(ms)**	**(ms)**	**(mT**/**m)**	**(s**/**mm** ^2^ **)**	**Nr**	**(ms)**	**(ms)**	**(mT**/**m)**	**(s**/**mm** ^2^ **)**
1	22	49	61	50	25	22	58	58	300
2	22	49	86	100	26	22	58	95	800
3	22	49	192	500	27	22	58	190	3,200
4	22	49	285	1,100	28	22	58	275	6,700
5	40	67	63	100	29	40	72	59	600
6	40	67	100	250	30	40	72	100	1,700
7	40	67	200	1,000	31	40	72	200	6,850
8	40	67	289	2,100	32	40	72	292	14,550
9	60	87	63	150	33	60	92	34	300
10	60	87	103	400	34	60	92	100	2,650
11	60	87	199	1,500	35	60	92	200	10,500
12	60	87	290	3,200	36	60	92	292	22,350
13	80	107	63	200	37	80	112	61	1,300
14	80	107	99	500	38	80	112	100	3,550
15	80	107	201	2,050	39	80	112	200	14,150
16	80	107	291	4,300	40	80	112	292	30,200
17	100	127	63	250	41	100	132	60	1,600
18	100	127	101	650	42	100	132	100	4,450
19	100	127	200	2,550	43	100	132	200	17,850
20	100	127	291	5,400	44	100	132	292	38,050
21	120	147	63	300	45	120	152	60	1,950
22	120	147	99	750	46	120	152	100	5,350
23	120	147	199	3,050	47	120	152	200	21,500
24	120	147	291	6,500	48	120	152	292	45,900

*Note:* We provide signal for the parts of protocol marked in black. In red is the protocol for which the signal needs to be predicted.

### 2.2 Post‐processing

All post‐processing was performed using Software Library (FSL).^56^ The DW images were corrected for eddy current distortions separately for each combination of δ and Δ using FSL's E d d y module (www.fmrib.ox.ac.uk/fsl/eddy) with its default settings. The images were then co‐registered using FSL's F n i r t package. As the 48 shells were acquired across a wide range of TEs, over two days, we chose to proceed in two steps. First, within each quarter of the dataset (different day, different δ) we registered all the b=0 images together. We then applied these transformations to their intermediary DW images, using a trilinear resampling interpolation. The second stage involved co‐registering the four different quarters. To help the co‐registration, especially between the two days images that required some through‐plane adjustment as well, we omitted areas of considerable eddy‐current distortions by reducing the number of slices from 20 to 5 (i.e. leaving two images either side of the mid‐sagittal plane) and reducing the in‐plane image size to 75×80.

### 2.3 Training and testing data

**Figure 1:**
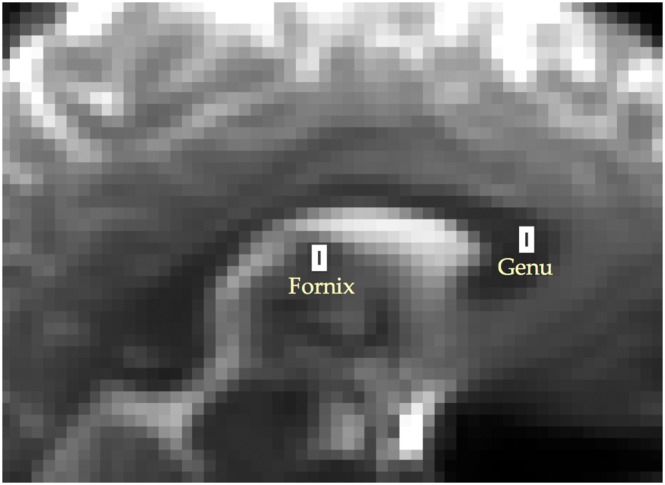
We only consider two ROIs, each containing six voxels from the genu in the corpus callosum, where the fibres are approximately straight and parallel, and from the fornix, where the configuration of fibres is more complex

The data for this work originated from two regions of interest (ROIs), each containing 6 voxels (see Figure 1). The first ROI was selected in the middle of the genu in the corpus callosum, where the fibres are mostly straight and coherent. The second ROI's fibre configuration is more complex: it lies in the body of fornix, where two bundles of fibres bend and bifurcate.

The dataset was split into two parts: the training dataset and the testing dataset. The training dataset was fully available for the challenge participants. The testing dataset was retained by one of the organizers (UF). The DW signal of the training dataset (36 shells, with acquisition parameters shown in black in Table 1) was provided together with the gradient scheme on the challenge website (http://cmic.cs.ucl.ac.uk/wmmchallenge/). This data was used by the participants to estimate their DW‐MRI model parameters. The signal attenuation in the testing dataset (12 shells, with acquisition parameters shown in red in Table 1) was kept unseen. It contained one shell, chosen at random, from each TE‐specific set of four shells (i.e of the same combination of δ and Δ). The challenge participants were then asked to predict the signal for the corresponding gradient scheme. They were free to use as much or as little of the training data provided as they wished to predict the signal of the test dataset for the six voxels in each ROI.

**Figure 2:**
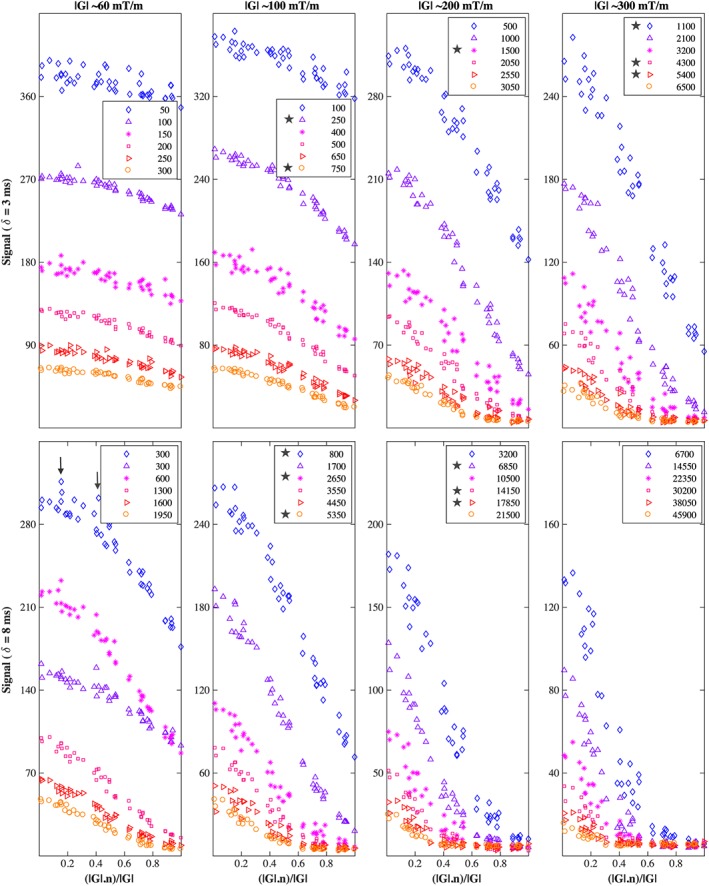
Diffusion‐weighted signal from the genu ROI, averaged over the six voxels. Across each column and row, the signal pertains to one of the gradient strengths or pulse times δ used; in each subplot, the six shells shown in different colours are Δ‐specific, increasing in value (22, 40, 60, 80, 100, 120 ms) from top to bottom. Inside the legend, the b value is in s/mm^2^ units; here, the HARDI shells kept for testing are those marked with a star; the remaining shells comprise the training data. On the x‐axis is the cosine of the angle between the applied diffusion gradient vector **G** and the fibre direction **n**. Some models in this study omit data outliers; two such data points are shown in the bottom‐left subplot with vertical arrows — obviously each model has its own criteria for determining the outliers

Figure 2 shows the DW signal attenuation for each shell in the genu dataset, with stars in the legend indicating which shells were left out for testing. In this plot, a small number of data appear as ‘outliers’ (two such data are shown with arrows in the bottom‐left subplot of Figure 2). Specifically, we counted about 10 of them among more than 4812 measurements, most of them being in the b=300 s/mm^2^ shell. Since these outliers appear to be specific to the b=300 s/mm^2^ shell and are not in other shells with similar b value, we attribute them to a momentary twitching of the subject rather than more systematic effects, such as perfusion.

**Figure 3:**
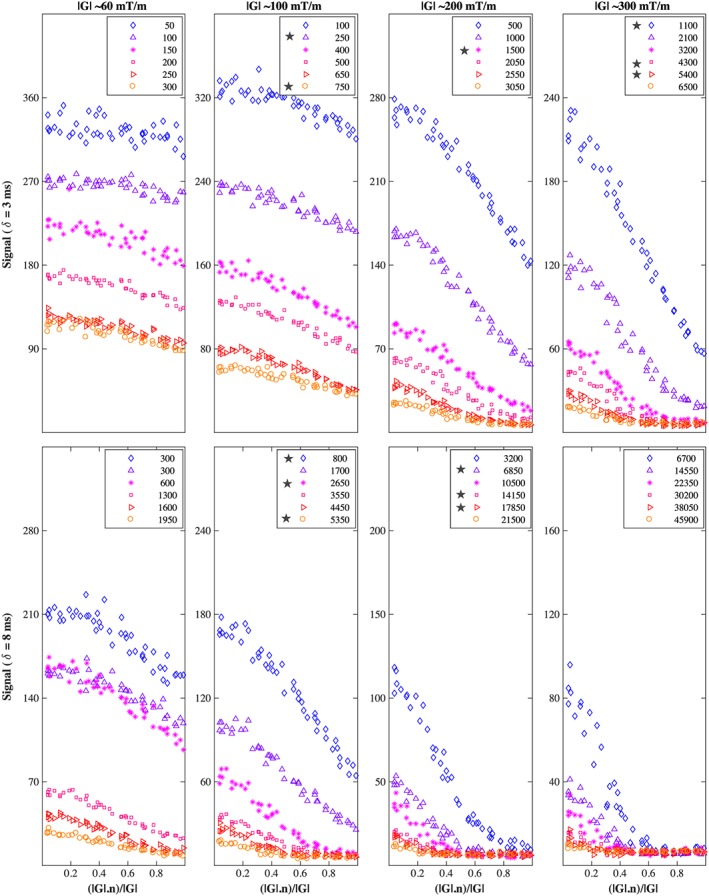
Diffusion‐weighted signal from the fornix ROI, averaged over the six voxels. The legend's b value is in s/mm^2^ units. Testing shells are marked with a star. On the x‐axis is the cosine of the angle between the applied diffusion gradient vector **G** and the fibre direction **n**

Similarly, Figure 3 shows the signal for the fornix region, with the signal over the six voxels averaged out.

### 2.4 Model ranking

Models were evaluated and ranked based on their ability to predict the unseen DW signal accurately. Specifically, the metric used was the sum of square differences between the hidden signal and the predicted signal, corrected for Rician noise:^57^

(1) $$SSE=\frac{1}{N}\sum_{i=1}^{N}\frac{{\left({\tilde{S}}_{i}-\sqrt{S_{i}^{2}+\sigma^{2}}\right)}^{2}}{\sigma^{2}}$$

where *N* is the number of measurements, ${\tilde{S}}_{i}$ is the *i*th measured signal, *S*_*i*_ its prediction from the model and *σ* the noise standard deviation.

### 2.5 Competing models

Here we give a short summary of the competing models. Additionally, Table 2 provides a summary of their key characteristics. More details are included in the Appendix.

- *Ramirez‐Manzanares:* a dictionary‐based technique that accounts for multiple fibre bundles and models the distribution of tissue properties (axon radius, parallel diffusivity) and the orientation dispersion of fibres.
- *Nilsson:* a multi‐compartment model that models isotropic, hindered and restricted diffusion and accounts for varying (*T*_1_, *T*_2_) relaxation times for each compartment.^58^
- *Scherrer* a multi‐compartment model in which each compartment is modelled by a statistical distribution of 3‐D tensors.^16^
- *Ferizi_1_ and Ferizi_2_:* two three‐compartment models that account for varying *T*_2_ relaxation times for each compartment. As regards the intracellular compartment, Ferizi_1_ models the orientation dispersion by using dispersed sticks as one compartment; Ferizi_2_ uses a single radius cylinder instead.^42^
- *Poot:* a three‐compartment model comprising an isotropic diffusion compartment, a tensor compartment and a model‐free compartment in which an Apparent Diffusion Coefficient (ADC) is estimated for each direction independently. *T*_2_ relaxation times are also estimated for each compartment.^59^
- *Rokem:* a combination of the sparse fascicle model^43^ with restriction spectrum imaging^60^ that describes the signal arising from a multi‐compartment model in a densely sampled spherical grid, using L1 regularization to enforce sparsity.
- *Eufracio:* an extension of the Diffusion Basis Function (DBF) model that accounts for multiple *b*‐value shells.
- *Loya‐Olivas_1_ and Loya‐Olivas_2_:* two models based on the Linear Acceleration of Sparse and Adaptive Diffusion Dictionary (LASADD) technique. Loya‐Olivas_1_ uses the DBF signal model, while Loya‐Olivas_2_ uses a three‐compartment tissue model. The optimization uses linearized signal models to speed up computation and sparseness constraints to regularize.
- *Alipoor:* a model of fourth‐order tensors, corrected for *T*_2_‐relaxation across different shells. A robust LS fitting was applied to mitigate influence of outliers.
- *Sakaie:* a two‐compartment model of restricted and hindered diffusion with angular variation. A simple exclusion scheme based on the *b*=0 signal intensity was applied to remove outliers.
- *Fick:* a spatio‐temporal signal model to represent 3‐D diffusion signal simultaneously over varying diffusion time. Laplacian regularization was applied during the fitting.^61^
- *Rivera:* a regularized linear regression model of diffusion encoding variables. This is intentionally built as a simplistic model to be used as a baseline for model comparison.

**Table 2:** Summary of the various diffusion models evaluated. Tissue models are models that include an explicit description of the underlying tissue microstructure with a multi‐compartment approach. In contrast, signal models focus on describing the DW signal attenuation without explicitly describing the underlying tissue and instead correspond to a ‘signal processing’ approach

	Type of model	Nb of free param. (genu/fornix)	Models effect of *δ* and Δ	Noise assumption	Optimization algorithm	Outliers strategy	Special signal prediction strategy
R–Manzanares	Tissue	N/A	Yes	Gaussian	weighted‐LS	Yes	CV
					bootstrapping		
Nilsson	Tissue	< 12/12	Yes	Gaussian	LM	Yes	CV
Scherrer	Tissue	10/16	No	Gaussian	Bobyqa	Yes	No
Ferizi_1	Tissue	< 12/12	Yes	approx.‐Rician	LM	No	No
Ferizi_2	Tissue	< 10/10	Yes	approx.‐Rician	LM	No	No
Alipoor	Signal	17/17	No	Gaussian	weighted‐LS	Yes	No
Sakaie	Signal	N/A	No	Gaussian	nonlinear‐LS	Yes	No
Rokem	Tissue	∼20	No	Gaussian	Elastic net	No	CV
				+ Noise floor			
Eufracio	Tissue	7/7	No	Gaussian	bounded‐LS	No	No
					Lasso, Ridge		
Loya‐Olivas_1	Tissue	11	No	Gaussian	bounded‐LS	No	No
					& Lasso		
Loya‐Olivas_2	Tissue	11	No	Gaussian	bounded‐LS	No	No
Poot	Signal	103	No	Rician	LM‐like	No	No
Fick	Signal	475	Yes	Gaussian	Laplacian‐ reg‐LS	No	partial‐CV
Rivera	Signal	23	Yes	Gaussian	Weighted Lasso	Yes	CV

Abbreviations: LS=least‐squares, LM=Levenberg–Marquardt, CV=cross‐validation, reg=regularized

While the challenge organizers also had competing models (Ferizi_1_, Ferizi_2_ and Scherrer), only Ferizi had access to the hidden data. The hidden data were never used to tune the results of his models.

## 3 RESULTS

**Figure 4:**
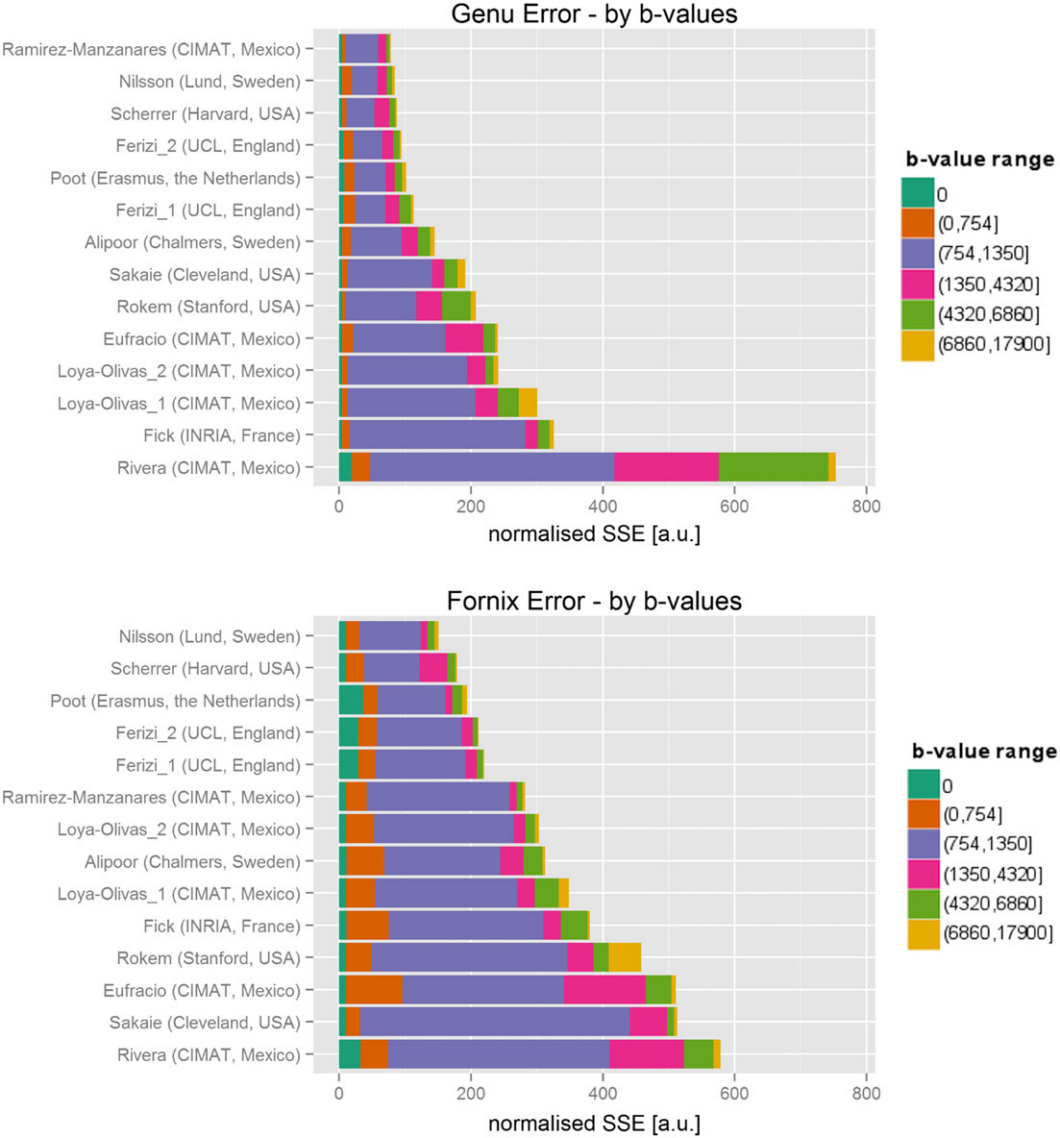
Overall ranking of models by sum‐of‐squared‐errors (SSE) metric over all voxels in genu (top) and fornix (bottom) ROIs. The colors represent different ranges of b‐value shells

Figure 4 shows the averaged prediction error in each ROI (top subplot is for the genu, bottom subplot is for the fornix) and the corresponding overall ranking of the participating models in the challenge. The first six models in the genu ranking performed similarly, each higher ranked model marginally improving on the prediction error. The prediction error clearly increased at a higher rate for the subsequent models. In the fornix dataset, the prediction error was higher than in the genu. For both datasets, the first six models were the same, albeit permuted. Most of the models performed similarly in terms of ranking in both genu and fornix cases, i.e. Nilsson (second in genu/first in fornix), Scherrer (third/second) and Ferizi_2 (fourth/fourth). Others performed significantly better in one of the cases, with Ramirez‐Manzanares (first/sixth) being the most notable.

Figure 4 also details the prediction error for different ranges of *b* values in the unseen dataset. Models inevitably vary in their prediction capabilities; some models perform better within a given *b*‐value range but are penalized more in another. Across the models, as the figure shows, the ranking between models was dominated by the signal prediction accuracy for *b* values between 750 and 1400s/mm^2^; specifically, the shell that has the largest weight on this error is the *b*=1100 s/mm^2^ one. The top‐ranking models, nevertheless, were better at predicting the signal for higher *b*‐value images as well. The prediction performance of lower *b*‐value images (<750s/mm^2^) in the genu was less consistent across ranks. For example, the models of Rokem and Sakaie outperformed most of the higher ranking models in this low *b*‐value range. The fornix is a more complex region than the genu, hence the performance across the shells is less consistent. In the fornix, the prediction errors were generally larger than in the genu across all *b* values for all models, except Rivera's, which showed the opposite effect. The prediction errors of the *b*=0 images were also larger than in the genu, especially for the highly ranked models of Poot and Ferizi. The prediction errors in other *b*‐value shells followed the overall ranking of the models more closely.

**Figure 5:**
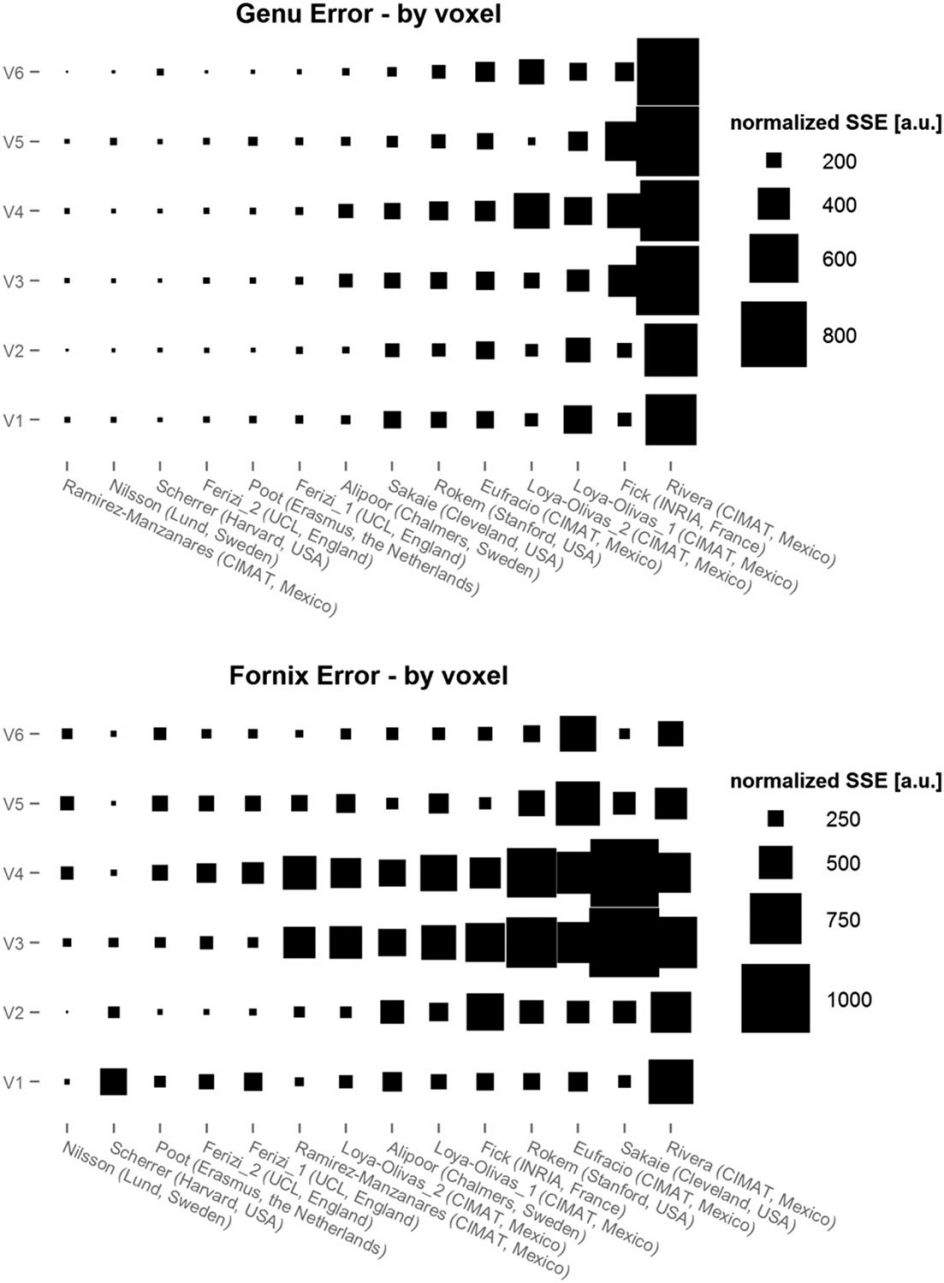
Sum‐of‐squared‐errors (SSE) per voxel for each model in genu and fornix. The size of rectangles represent the SSE value per voxel

Figure 5 shows the prediction error for each voxel independently. In the genu plot, the best performing models had high consistency of low prediction errors across all individual voxels. These were followed by the models with consistent larger prediction error in all voxels. Most of the lowest ranking models not only had largest prediction errors, they also showed large variations in prediction performance. For example, while the model of Loya‐Olivas_2_ was competitive in voxel 5, it ranked low due to large prediction errors in voxels 4 and 6. The results in the fornix show a lower consistency of prediction errors between the voxels than in the genu. Specifically, two voxels (3 and 4) showed substantially larger prediction errors and were likely responsible for much of the overall ranking.

**Figure 6:**
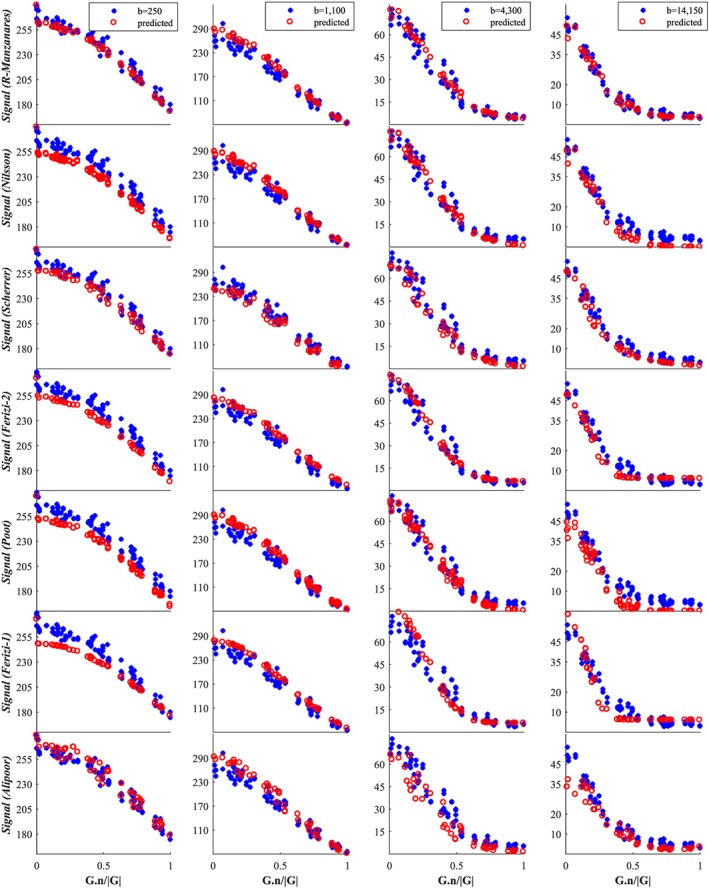
Genu signal for the group consisting of the best seven from 14 models. We show only four (of twelve) representative shells; these are shown by blue stars, while red circles denote the model‐predicted data. The best models are listed first. The x‐axis is the cosine of the angle between **G** and **n**

**Figure 7:**
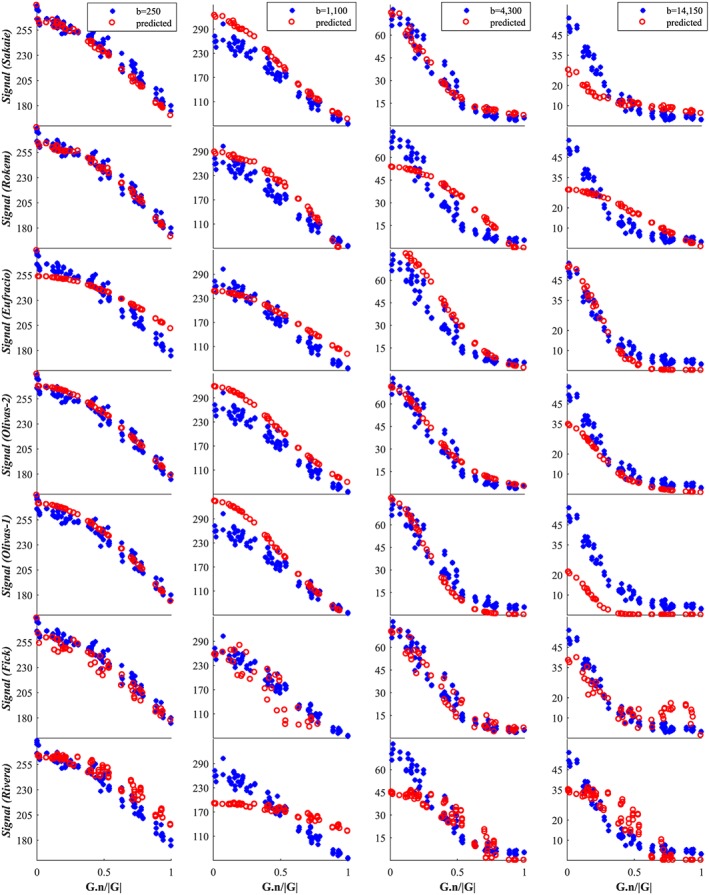
Genu signal for the second group of 14 models. Raw testing data are shown by blue stars, while red circles denote the model‐predicted data. The x‐axis is the cosine of the angle between **G** and **n**

**Figure 8:**
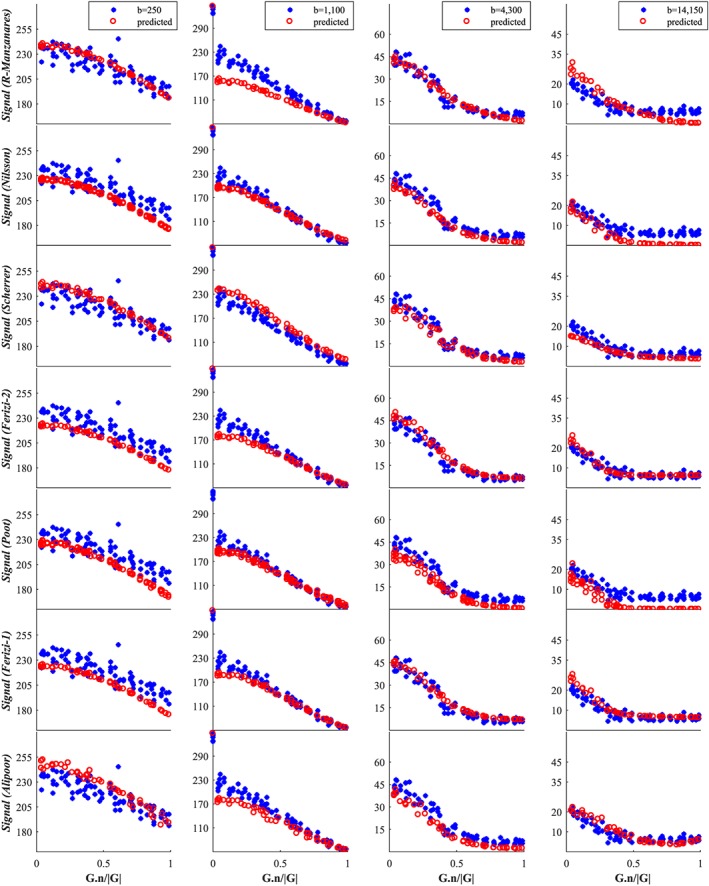
Fornix signal for the group consisting of the best 7 from 14 models. We show only four (of twelve) representative shells; these are shown by blue stars, while red circles denote model‐predicted data. The best models are listed first. The x‐axis is the cosine of the angle between **G** and **n**

**Figure 9:**
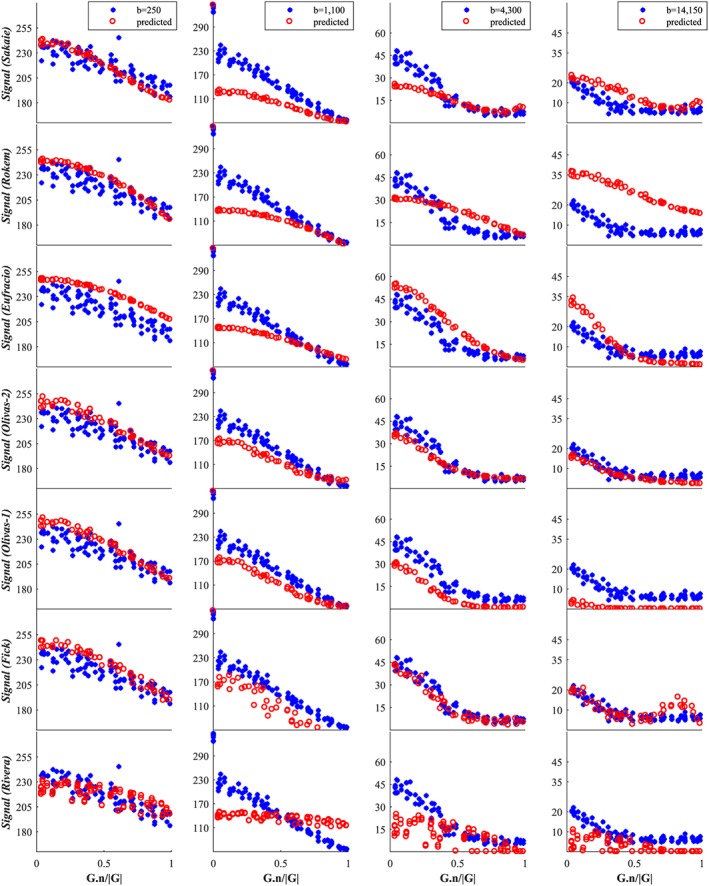
Fornix signal for the second group of 14 models. Raw testing data are shown by blue stars, while red circles denote the model‐predicted data. The x‐axis is the cosine of the angle between **G** and **n**

Finally, we report in Figures 6, 7, 8 and 9 an illustration of the quality of fit of each model to four representative shells, including the b=1100s/mm^2^ shell mentioned above; Figures 6 and 7 concern the genu data and Figures 8 and 9 are for fornix data.

## 4 DISCUSSION

The challenge set out to compare the ability of various kinds of models to predict the diffusion MR signal from WM over a very wide range of measurement parameters – exploring the boundaries of possible future quantitative diffusion MR techniques. The 14 challenge entries were a good representation of the many available models that are proposed in the literature. The acquired data aimed to cover the broadest spectrum of experimental parameters possible. The participating models use a variety of fitting routines and modelling assumptions, providing additional insight into the effects of algorithmic and modelling choices during parameter estimation. Although the set of methods tested is not sufficient to make a full comparison of each independent feature (diffusion model, noise model, fitting routine, etc.) and the number of combinations prohibits an exhaustive comparison, the results of the challenge do reveal some important trends.

In contrast with earlier model comparisons,^18^, ^43^, ^44^ the results provide new insight into which broad classes of model explain the signal best and what features of the estimation procedure are important. This information is very timely, as recent model‐based diffusion MRI techniques, such as NODDI,^15^ SMT,^17^, ^40^ DIAMOND,^16^ DKI^62^ and LEMONADE,^63^ are starting to become widely adopted in clinical studies and trials. Despite their success, intense debate continues in the field about applicability of different models and fitting routines.^64^, ^65^ The insights from this challenge provide key pointers to the important features of the next‐generation of front‐line imaging techniques of this type. Moreover, the data and evaluation routines remain available to form the basis of an expanding ranking of models and fitting routines and a benchmark for future model development.

### 4.1 Main conclusions

The first insight is on the type of model used. Signal models do not necessarily outrank tissue models; indeed, using our dataset, models of the signal (Alipoor, Sakaie, Fick, Rivera) ranked on average lower than models of the tissues, despite their theoretical ability to offer more flexibility in describing the raw signal. This is quite surprising, as the current perception within the field is that, generally, we can capture the signal variation much better through a functional description of the signal (signal models) rather than via a biophysical model of the tissue (tissue models). The former generally consist of bases of arbitrary complexity, whereas the latter are generally very parsimonious models that rely on extremely crude descriptions of tissue (e.g. white matter as parallel impermeable cylinders). The results suggest that the flexibility of signal models can rapidly lead to overfitting. However, the tissue models can explain the signal relatively well even with just a few parameters (compare the quality‐of‐fit plots of the Rivera model in Figure 7 with the signal prediction of the top models in Figure 6: the higher the *b* value, the worse the prediction of the linear signal model). Certain underlying assumptions may cause the signal models to perform less well than expected. For example, they are often designed to work with data with a single diffusion time and do not generalize naturally to incorporate the additional dimension (although see Fick et al.^61^ for some steps towards generalization). Many of the tissue models, on the other hand, naturally account for finite *δ*, varying diffusion times and gradient strength (e.g. the Ramirez‐Manzanares, Nilsson and Ferizi models in our collection). We cannot draw any conclusion about the benefits of an adjustable number of parameters in a model, because of the limited number of models in our study that do this and because the models differ in a range of other aspects.

The second insight concerns the choice of noise modelling. Despite the fact that SNR at *b*=0 and *T* *E*=152 ms falls to about 6, use of the Rician noise model does not appear to be a significant benefit in predicting unseen signal; here, however, we do not investigate the effect on estimated model parameters, which may still benefit from the more accurate noise model. In this challenge, most participants used non‐linear least‐squares or maximum‐likelihood optimization. Additional regularization of the objective function (Eufracio & Rivera/Lasso, Rokem/Elastic Net, Fick/Laplacian) appeared to have little benefit over non‐regularized optimization.

The third observation is about removing signal outliers. Five of the eleven models preprocessed the training data by clearing out outliers, including the top two models. We tried this procedure with two good models that did not use such a procedure, Ferizi_1_ and Ferizi_2_, and observed that it did not affect the ranking, though it did improve the prediction error marginally. This is understandable, considering the relatively little weight these apparent outliers have on the total number of measurements (10 points from a 4812‐strong dataset). Additionally, specific strategies for predicting the signal, e.g. bootstrapping or cross‐validation, as used by the top two models of Ramirez‐Manzanares and Nilsson, may also help the model ranking.

### 4.2 Limitations and future directions

Although this challenge provides several new insights into the choice of model and fitting procedure for diffusion‐based quantitative imaging tools, it has a number of limitations that future challenges might be designed to address. One limitation of the study is that we use a very rich acquisition protocol that is not representative of common or clinical acquisition protocols. In particular, we cover a very wide range of *b* values and the data acquisition (protocol) we use consists of many TEs, unlike many other multi‐shell diffusion datasets that use a fixed TE. As stated in the Introduction, our intention is to sample the measurement space as widely as possible to support the most informative models possible. Varying the TE makes it possible to probe compartment‐specific *T*_2_ (the decay of which Ferizi et al.^42^ finds to be monoexponential at the voxel level), an investigation that would be impossible with a single TE. However, the good performance of DIAMOND also shows that a model with fixed *δ* and Δ can still capture the signal variation in multi‐TE datasets and that, while the majority of the full data was ignored in each of the reconstructions, its prediction error compared favourably with other techniques.

We use the unique human Connectome scanner^51^ to acquire a dataset with gradients of up to 300mT/m, which is not readily available in most current MR machines. However, previous preclinical work by Dyrby et al.^13^ suggests that high diffusion gradients enrich the signal, which helps model fitting and comparison. Future challenges might be designed that focus on explaining the signal and estimating parameters from data more typical of clinical acquisitions.

Assessing the prediction performance on unseen data as in this challenge is different from assessing the fitting error: it implicitly penalizes models that overfit the data. However, since most of the missing shells lie in between other shells (in terms of *b* values, TEs, etc.), the quality of signal extrapolation was not assessed. We get a glimpse of this from Figure 4, where the SSE is unevenly distributed between the *b* values. Here, the shell that bore the largest error is the *b*=1100 s/mm^2^ one; see also Figures 6 and 7. Of all ‘unseen’ shells, this shell combines the lowest Δ and highest |*G*|, placing it on the edge of the range of the measurement space sampled. Such a *b*‐value shell combines high signal magnitude with high sensitivity, i.e. the gradient of signal against *b*‐value is highest in this range, which makes it hard to predict. (We stress that this observation is in the context of the wider multi‐shell acquisition, and is not to be seen in isolation for its potential impact on single‐shell acquisition methods.) On the other hand, the variability of prediction errors in the *b*<750 s/mm^2^ range could arise from the varying sensitivity of different models to the free water component, which is challenging to estimate as it can easily be confounded with hindered water, or physiological effects, which are mostly observable in this low *b*‐value range. Future work can take this further, by selecting unseen shells outside the min–max range of experimental parameters. This is likely to penalize more complex models that overfit the data even more strongly.

We did not take into account the computational demand of each model, and this might limit the generalization of the results. Models that use bootstrapping generally have a higher computational burden and may not be feasible for large datasets, e.g. whole brain coverage.

The dataset used in this challenge is specific to one subject who underwent a long‐duration acquisition, which adds to the question of generalizability. The subsequent preprocessing of the data is also a factor to bear in mind: the registration of two 4h datasets, across such a broad range of echo times, poses its own challenges for certain non‐homogenous regions in the brain, such as the fornix (compared with, for example, the relatively large genu). Thus the results may be somewhat subject‐specific and may be affected by residual alignment errors.

Another limitation is that we only look at isolated voxels inside the corpus callosum and the fornix. Questions still remain about which models are viable even in the most coherent areas of the brain with the simplest geometry, so we believe our focused challenge on well‐defined areas is an informative first step necessary before extending the idea to the whole of the white matter, which would make for an extremely complex challenge. We note, however, recent work by Ghosh et al.^66^ that illustrates such an approach with Human Connectome Project (HCP) data.

We focused here on comparing models based on their ability to predict unseen data. Although models that reflect true underlying tissue structure should explain the data well, we cannot infer in general that models that predict unseen data better are mechanistically closer to the tissue than those that do not. As we discuss in the Introduction, the main power of evaluating models in terms of prediction error is to reject models that cannot explain the data. Thus, while the identification of parsimonious models that explain the data certainly has great benefit, further validation is necessary through comparison of the parameters that they estimate with independent measurements, e.g. obtained through microscopy (our challenge makes no attempt to assess the integrity of parameter estimates themselves, but future challenges might use such performance criteria). Models can be evaluated to some extent by sanity checking the realism of their fitted parameter values, as in for example Jelescu et al.^64^ or Burcaw et al.^67^ However, obtaining accurate ground‐truth values for quantitative evaluation remains a hard and yet unsolved problem for diffusion MRI in general. In particular, histology can only roughly approximate the in vivo ground truth and introduces its own set of challenges in sample preparation, acquisition and biophysical interpretation.^12^, ^13^, ^65^, ^68^, ^69^, ^70^, ^71^ This challenge highlights the need for improved model comparison and validation methods.

## 5 CONCLUSION

Challenges such as this have great value in bringing the community together and provide an unbiased comparison of wide‐ranging solutions to key data‐processing problems. They raise new insights and ideas, motivating more directed future studies. The data are publicly available for others to use, with more details of the dataset given on the Challenge website at http://cmic.cs.ucl.ac.uk/wmmchallenge/. On this website, an up‐to‐date ranking of the models will be available, where additional models can be added after the publication of the article and where the community will be able to evaluate further the impact of noise correction, compartment‐specific *T*_2_ estimation, inter‐class model assumptions, e.g. tissue versus signal models, or indeed intra‐class model assumptions, e.g. whether cylinders or sticks are optimal models for the given dataset.^42^ This will provide an important benchmark for future models and parameter estimation routines.

## COMPETING MODELS

### A.1 Tissue models

#### A.1.1 Ramirez‐Manzanares (CIMAT, Mexico): Empirical Diffusion‐and‐Direction Distributions (ED^3^)

This work builds on the statistical modelling of the apparent diffusion coefficient^72^ and tackles the modelling of axon fibre dispersion in single^15^, ^73^ and multiple fibre bundle cases. The method estimates empirically (rather than imposes) the distribution of tissue properties (axon radius, parallel diffusion, etc.), as well as the orientational distribution of the bundles. The general framework is as follows:

- estimation of mean principal diffusion directions (PDD) per axon bundle;
- selection of a dense set of orientationally focused basis directions that capture the discrete non‐parametric fibre dispersion;
- design of a dictionary of intra/extracellular synthetic DW signals, which are precomputed along the basis directions (see the DBF method in Ramirez‐Manzanares et al.^74^);
- computation of the size compartments per diffusion atom of the dictionary (model fitting).

The PDDs are estimated from the Diffusion Tensor (DT) (single bundle case) and DBF^74^ (complex structure cases). The 120 orientations closest to the PDDs are selected from a set of 1000 evenly distributed orientations. The intra‐axonal signals *S* ^*i*^ are precomputed from the model in Van Gelderen et al.^75^ for restricted diffusion within a cylinder with radius R=1,2,⋯,10μm and parallel diffusion $d_{∥}=1,1.1,\text{⋯},2.1$μm^2^/ms. The extra‐axonal signals are generated as follows: *S* ^*e*^ from *zeppelins* with combinations of parallel and radial diffusion, $d_{∥}=1,1.1,\text{⋯},2.5$μm^2^/ms and $d_{⊥}=2,3,\text{⋯},8$μm^2^/ms; isotropic diffusion compartment signals $S_{i}^{\text{iso}}=\exp^{-q\tau d_{\text{iso}}}$ for $d_{\text{iso}}=2,2.1,2.2,\text{⋯},4$μm^2^/ms; and the *dot* signal, which takes into account static proton density. The values of the dictionary atoms above were tuned by cross‐validation.^76^ The size compartments *β*⩾0 computed in the weighted non–negative LS formulation,

(A1) $$\begin{array}{c} {\left|\left|W[S-S_{0}^{TE}(\sum_{i=1}^{N_{i}}\beta_{i}^{i}S_{i}^{i}-\sum_{j=1}^{N_{e}}\beta_{j}^{e}S_{j}^{e}-\sum_{k=1}^{N_{\text{iso}}}\beta_{k}^{\text{iso}}S_{k}^{\text{iso}}+\beta_{\text{dot}})]\right|\right|}_{2}^{2} \end{array}$$

indicate the atoms that explain the signal; the *W* weights are proportional to SNR. Overfitting is reduced by a *bootstrap* 77 procedure.

The cross‐validation experiments indicate that the reconstructions given by the robust fitting of this rich multi‐compartment diffusion dictionary allow us to predict accurately non‐acquired MR signals for different machine protocols. This is of most interest in the development of methods able to detect the complex microstructure heterogeneity associated with the different compartments within the voxels. The atoms with coefficients *β*>0 depict the empirical distributions and their orientations indicate non‐parametrical bundle–dispersion configurations (such as fanning or radially symmetrical). The recovered distributions reveal, for instance, an axon radius of between 1 and 4μm. One should take into account, however, that, since the heterogeneous intra/extra‐axonal *T*_2_ relaxation feature is not modelled explicitly, the method may compensate for *T*_2_ variations by using, for instance, large isotropic *d*_iso_ coefficients to fit the signal accurately. For this reason, a direct interpretation of the fitted parameters may be misleading. The use of more specific models is a part of ongoing work.

#### A.1.2 Nilsson (Lund, Sweden): multi‐compartment model outlier rejection and separate fitting of b_0_data

This multiple compartment model was developed specifically for the ISBI WM challenge and built up by relaxation‐weighted and time‐dependent diffusion tensors according to

(A2) $$S=S_{0}\sum_{i}w_{i}\;e^{-B:D_{i}}e^{-{\text{TE/T2}}_{i}}(1-e^{-\text{(TR}-\text{TE/2)/T}1_{i}})$$

where $B=b{\vec{n}}^{⊗2}$ and *b*=(*γ* *δ* *g*)^2^ *t*_d_. The diffusion time *t*_d_ was corrected for rise times (*ξ*) according to *t*_d_=Δ−*δ*/3+*ξ* ^3^/30*δ* ^2^−*ξ* ^2^/6*δ*. Each component was also described by a weight (*w*_*i*_) and relaxation times (T1_*i*_ and T2_*i*_). The model featured three types of component, with either isotropic, hindered or restricted diffusion. Diffusion in the isotropic component was modelled by a single diffusion coefficient. The hindered and restricted components were modelled by cylinder‐symmetric tensors described by axial and radial diffusivities together with the polar and azimuth angles. In the restricted component, the apparent diffusion coefficient of the radial component depended on *δ* and Δ, as well as on the cylinder radius, according to Van Gelderen et al.^78^

Three modifications were performed to this very general model. First, to accommodate for potential bias in the *b*_0_ images (which was the case for fornix data, where deviations of up to 20*σ* was observed), the prediction for *b*_0_ data was obtained from the median of all signals acquired with identical TE instead of from Equation A2. Second, opposite direction acquisitions were rescaled by a free model parameter, in order to allow for potential gradient instabilities inducing differences between the directions and their opposite directions. Third, models were generated dynamically during fitting by randomly selecting up to four hindered components and up to three restricted components. One isotropic component was always included.

The model was first fitted to half of the diffusion‐weighted data (randomly selected), after which outliers were rejected (>2.5*σ*). Thereafter a second fit was performed. Both fit steps assumed Gaussian noise and utilized the ‘lsqcurvefit’ function in Matlab. The procedure was repeated 100 times for different randomly generated models.

To prepare for submission of the results, only the models that best predicted the hidden half of the data were selected, after which the median of the selected predictions was used for the final prediction.

#### A.1.3 Scherrer (Harvard, USA): distribution of anisotropic microstructural environments in diffusion compartment imaging (DIAMOND)

DIAMOND models the set of tissue compartments in each voxel by a finite sum of unimodal continuous distributions of diffusion tensors. This corresponds to a hybrid tissue model that combines biophysical and statistical modelling. As described by Scherrer et al.,^16^ the DW signal *S*_*k*_ for a gradient vector **g**_*k*_ and *b* value *b*_*k*_ is modelled by

$$S_{k}=S_{0}\left[\overset{N}{\underset{j=0}{\sum }}f_{j}{\left(1+\frac{b_{k}g_{k}^{T}D_{j}^{0}g_{k}}{\kappa_{j}}\right)}^{-\kappa_{j}}\right]$$

where *S*_0_ is the non‐attenuated signal, *N* is the number of compartments, *f*_*j*_ the relative fraction of occupancy of the *j*th compartment and *κ*_*j*_ and $D_{j}^{0}$ are respectively the concentration and expectation of the *j*th continuous tensor distribution. DIAMOND enables assessment of compartment‐specific diffusion characteristics such as the compartment FA (cFA), compartment RD (cRD) and compartment MD (cMD). It also provides a novel measure of microstructural heterogeneity for each compartment.

The estimation of a continuous distribution of diffusion tensors requires DW data acquired with the same timing parameters *δ* and Δ.^16^ To compare DIAMOND with other models in this dataset, we fitted one DIAMOND model separately for each {*δ*, Δ} group (i.e. for each TE group), leading to 12 DIAMOND models. One shell was missing in each TE group; we predicted its signal using the corresponding DIAMOND model. The model estimation was achieved as follows. We first computed the mean and standard deviation of *S*_0_( $\mu_{S_{0}}$ and $\sigma_{S_{0}}$) within each TE group and discarded DW signals with intensity larger than $\mu_{S_{0}}+3\sigma_{S_{0}}$(simple artefact correction). We then estimated DIAMOND parameters as described in Scherrer et al.,^16^ considering Gaussian noise and cylindrical anisotropic compartments. For the genu, we considered a model with one freely diffusing and one anisotropic compartment; for the fornix, we considered a model with one freely diffusing compartment and two anisotropic compartments.

#### A.1.4 Ferizi_1 and Ferizi_2 (UCL, England)

This submission uses two three‐compartment models, as described in previous studies.^39^, ^41^ These models consist of (1) either a Bingham distribution of sticks or a cylinder for the intracellular compartment; (2) a diffusion tensor for the extracellular compartment; (3) an isotropic cerebrospinal fluid (CSF) compartment. The *T*_2_ relaxation element is fitted beforehand to the (variable echo time) *b*=0 measurements. The signal model is as follows:

(A3) $$S=\tilde{S_{0}}[f_{i}\exp(-\frac{TE}{T_{2}^{i}})S_{i}+f_{e}\exp(-\frac{TE}{T_{2}^{e}})S_{e}+f_{c}\exp(-\frac{TE}{T_{2}^{c}})S_{c}]$$

where *f*_i_, *f*_e_ and *f*_c_ are the weights of the intracellular, extracellular and third normalized compartment signals *S*_i_, *S*_e_ and *S*_c_, respectively; the values of compartmental *T*_2_ are indexed similarly; $\tilde{S_{0}}$ is the proton density signal (which is TE‐independent and obtained from fitting to the *b*=0 signal). These models emerged from previous studies.^41^, ^44^ Here, however, a single white matter *T*_2_ and separate compartmental diffusivities are additionally fitted.

There is a two‐stage model fitting procedure. The first step estimates the *T*_2_ decay rate of tissue separately in each voxel, by fitting a bi‐exponential model to the *b*=0 intensity as a function of TE, in which one component is from tissue and the other from CSF. A preliminary analysis of voxels fully inside WM regions shows no significant departure from mono‐exponential decay; equal *T*_2_ values are then assumed within the intra and extracellular compartments. When fitting the bi‐exponential model, the value of *T*_2_ in CSF is fixed to 1000 ms (a more precise value of CSF is unlikely to be estimated with this protocol). Thus, for each voxel, the volume fraction of CSF, $\tilde{S_{0}}$ and *T*_2_ of the tissue are estimated. These three estimates are then fixed for all the subsequent model fits. Then, each model is fitted using the Levenberg–Marquardt algorithm with an offset Gaussian noise model. The model parameters obtained were similar to earlier estimates obtained using the full dataset,^42^ differing by between 5–10% from the original.

#### A.1.5 Poot (Erasmus, the Netherlands)

This submission uses a three‐compartment model, with for each compartment a different complexity of the diffusion model and an individual *T*_2_ value. This model was developed specifically for the ISBI WM challenge and is the result of iteratively visualizing different projections of the residuals and trying to infer the maximum complexity that the rich data supports.

The first compartment models isotropic diffusion and, through the initialization procedure, it captures the fast diffusion components. The second compartment is modelled by a second‐order (diffusion) tensor and models intermediate diffusion strengths. The third compartment is model‐free, as the ADC is estimated for each direction independently. Each compartment additionally has an individual *T*_2_ value and signal intensity at *b*=0, *T* *E*=0 (which could easily be translated into volume fractions). Hence, the complete model of a voxel in image *j* is given by

(A4) $$S_{j}(\theta )=\overset{3}{\underset{i=1}{\sum }}A_{i}e^{-TE_{j}R_{2,i}}e^{-b_{j}ADC_{j,i}}=\overset{3}{\underset{i=1}{\sum }}e^{M_{i,j}\theta }$$

where *S*_*j*_ is the predicted signal intensity of image *j*, *A*_*i*_ is the non‐diffusion weighted signal intensity of compartment *i* at zero *T* *E*, *T* *E* is the echo time, *R*_2_ is the reciprocal of the *T*_2_ relaxation time of compartment *i*, *b*=(Δ−*δ*/3)*δ* ^2^|*G*|^2^ *γ* ^2^, with *γ*=42.5781 MHz/T, *A* *D* *C*_*j*,1_=*c*, $ADC_{j,2}=g_{j}^{T}Dg_{j}$, $ADC_{j,3}=dh_{j}^{T}$, where ***d*** is a vector with the ADC value of each orientation group and ***h***_*j*_ is a vector that selects the orientation group to which image *j* belongs (90 groups in total). Note that ***h***_*j*_ has at most one non‐zero element and that element has a value of one. As displayed in the rightmost part of Equation A4, the model can be written as a multiplication of matrices ***M***_*i*_, containing all rows ***M***_*i*,*j*_, with $\theta =[\ln A_{1}$, *R*_2,1_, *c*, $\ln A_{2}$, *R*_2,2_, *D*_11_, *D*_12_, *D*_13_, *D*_22_, *D*_23_, *D*_33_, $\ln A_{3}$, *R*_2,3_, ***d***]^T^, which combines all 103 parameters into a single parameter vector. All parameters are estimated simultaneously from the 3311 measurements provided per voxel by a maximum‐likelihood estimator that assumes a Rician distribution of the measurements and simultaneously optimizes the noise level.^59^ Finally, the signal intensities of the ‘unseen’ data are predicted by substituting the estimate into Equation A4.

#### A.1.6 Rokem (Standford, USA): a restriction‐spectrum sparse fascicle model (RS‐SFM)

The sparse fascicle model (SFM)^43^ is a member of the large family of models that account for the diffusion MRI signal in the white matter as a combination of signals due to compartments corresponding to different axonal fibre populations (fascicles) and other parts of the tissue. Model fitting proceeds in two steps. First, an isotropic component is fitted. We model the effects of both the measurement echo time (TE) and the measurement *b* value on the signal. These are fitted as a log(TE)‐dependent decay with a low‐order polynomial function and a *b*‐value‐dependent multi‐exponential decay (also including an offset to account for the Rician noise floor). The residuals from the isotropic component are then deconvolved with the perturbations in the signal due to a set of fascicle kernels, each modelled as a radially symmetric (*λ*_2_=*λ*_3_) diffusion tensor. The putative kernels are distributed in a dense sampling grid on the sphere. Furthermore, restriction spectrum imaging (RSI)^60^ is used to extend the model, by adding a range of fascicle kernels at each sampling point, with different axial and radial diffusivities, capturing diffusion at different scales. To restrict the number of anisotropic components (fascicles) in each voxel and to prevent overfitting, the RS‐SFM model employs the Elastic N et algorithm (EN),^79^ which applies a tunable combination of L1 and L2 regularization on the weights of the fascicle kernels. We used elements of the SFM implemented in the *dipy* software library^80^ and the EN implemented in *scikit‐learn*.^81^ In addition, to account for differences in SNR, we implemented a weighted least‐squares strategy, whereby each signal's contribution to the fit was weighted by its TE, as well as the gradient strength used. EN has two tuning parameters, determining (1) the ratio of L1‐to‐L2 regularization and (2) the weight of the regularization relative to the least‐squares fit to the signal. To find the proper values of these parameters, we employed *k*‐fold cross‐validation,^43^ leaving out one shell of measurement in each iteration for cross‐validation. We determined that the tuning parameters with the lowest Least Squares Error (LSE)^18^ provide an almost‐even balance of L1 and L2 penalty with weak overall regularization. Because of the combination of a dense sampling grid (362 points distributed on the sphere) and multiple restriction kernels (45 per sampling point), the maximal number of parameters for the model is approximately 16 300, more than the number of data points. However, because regularization is employed, the effective number of parameters is much smaller, resulting in an active set of approximately 20 regressors.^82^ We have made the code to reproduce our results fully available at https://arokem.github.io/ISBI2015.

#### A.1.7 Eufracio (CIMAT, Mexico): diffusion basis functions for multi–shell scheme

This model is based on the Diffusion Basis Functions (DBF) model,^74^ a discrete version of the Gaussian Mixture Model for the sphere: ${ŝ}_{i}=\sum_{j=1}^{m}\alpha_{j}\phi_{ij}+\epsilon$, with ${ŝ}_{i}=s_{i}/s_{0}$, $\phi_{ij}=\exp(-bq_{i}^{T}T_{j}q_{i})$ and $T_{j}=(\chi_{1}v_{j}v_{j}^{T}+\chi_{2}I)$. The DBF model is reformulated by substituting *ϕ*_*i**j*_ and *T*_*j*_: ${ŝ}_{i}=\sum_{j=1}^{m}\alpha_{j}\exp\left(-b_{i}\chi_{2}g_{i}^{T}g_{i}\right)\exp\left(-b_{i}\chi_{1}{\left(v_{j}^{T}g_{i}\right)}^{2}\right)+\epsilon$. The first exponential can be defined as a scale factor that depends on the *b* values, $\beta_{i}=\exp(-b_{i}\chi_{2}q_{i}^{T}q_{i})$. In this way, the *β*_*i*_ factors are associated with different *b* values, so the new model includes information for multi‐shell schemes. The coefficients *α* and the shell scale factor *β* are computed by solving the optimization problem:

(A5) $$\min_{\alpha ,\beta_{c}}\;f(\alpha ,\beta_{c};\lambda_{\alpha },\lambda_{\beta })=∥B\tilde{\Phi }\alpha -S{∥}_{2}^{2}+\lambda_{\alpha }∥\alpha {∥}_{1}+\lambda_{\beta }∥\beta_{c}^{0}-\beta_{c}{∥}_{2}^{2}\;\;\;\text{s.t.}\;1^{T}\alpha =1,\alpha ⩾0$$

where *B*= diag(*β*_*c*_),

$$\beta_{c}=\frac{1}{\#C}\sum_{i\in C}\exp(-b_{i}{\hat{\chi }}_{2}(q_{i}^{T}q_{i}))$$

and *C* is the set of indices grouped by different *b* values (#C is the number of elements in it). The regularization term weighted by *λ*_*α*_ demands sparseness and the term weighted by *λ*_*β*_ prevents overfitting. The problem in Equation A5 is solved in three steps. First, the active atoms are predicted (*α*_*i*_>0) with $\tilde{\alpha }={\mathrm{argmin}}_{\alpha }f(\alpha ,\beta_{c};\lambda_{\alpha },\lambda_{\beta })$. Second, the active atoms are corrected with $\alpha ={arg min}_{\{\alpha_{i}\}:{\tilde{\alpha }}_{i}>0}f(\alpha ,\beta_{c};0,\lambda_{\beta })$. Finally, the factors *β*_*c*_ are updated with $\beta_{c}={\text{argmin}}_{\beta_{c}}f(\alpha ,\beta_{c};\lambda_{\alpha },\lambda_{\beta })$. To solve each step, the active sets algorithm for quadratic programming is used.

To train the model for the WMM'15 data, Equation A5 is solved for each voxel with the training data to find the optimal weights *α*_*j*_ and scale factors *β*_*c*_ that best reproduce the training data. For this challenge, the *β*_*c*_ factors are grouped by the 36 training shells and the method parameters are set by hand: *λ*_*α*_=0.5, *λ*_*β*_=0.02, *χ*_1_=9.5×10^−4^and *χ*_2_=5×10^−5^. To predict the unseen signal at each voxel, the reformulated model is used with the optimal weights *α*_*j*_ and the 12 scale factors for the unseen *β*_*c*_ are calculated by interpolation with the 36 optimal *β*_*c*_ of the training data.

#### A.1.8 Loya‐Olivas_1 and Loya‐Olivas_2 (CIMAT, Mexico): Linear Acceleration of Sparse and Adaptive Diffusion Dictionary (LASADD)

LASADD is a multi–tensor based technique to adapt dynamically the diffusion functions (DFs) dictionary to a DW–MRI signal.^83^, ^84^ The method changes the size and orientation of relevant diffusion tensors (DTs). The optimization algorithm uses a special DT expression and assumptions to reduce the computational cost.

The one‐compartment version (LASADD–1C) is based on a DBF multi–tensor model:^74^ $s_{i}^{*}=\sum_{j=1}^{n}\alpha_{j}\phi_{i,j}$, where $s_{i}^{*}=s_{i}/{s_{0}}_{i}$, $\phi_{i,j}=\exp\left(-b_{i}g_{i}^{T}T_{j}g_{i}\right)$, *α*_*j*_>0 and $\sum_{j=1}^{n}\alpha_{j}=1$. LASADD expresses the DT as

(A6) $$T_{j}={\chi_{1}}_{j}v_{j}v_{j}^{T}+{\chi_{2}}_{j}I,$$

where $\chi_{{\left\{1,2\right\}}_{j}}$ are scalars associated with the eigenvalues, **v**_*j*_ is the principal diffusion direction (PDD) and **I** is the identity matrix. The algorithm iterates three steps, like Aranda et al.:^85^, ^86^ Predict, Correct, and Generate, until convergence. Prediction selects the relevant DFs using LASSO to regulate the number to choose. Correction adjusts the volume fraction, size and orientation of the DTs. Taking advantage of the DT expression and Taylor first‐order series approximation of the exponential, the optimizations are reduced to bounded least‐squares problems, which are solved by a Projected Gauss–Seidel scheme. Generation controls the overestimation of fibres by adding to the basis the DTs resulting from combining two and three DFs for the new iteration.

An extra refinement to the computed results, named LASADD–3C, splits each detected DF into three compartments:^87^ intracellular (IC), extracellular (EC) and CSF. The multi‐tensor model is $s_{i}^{*}=\sum_{j=1}^{n}\alpha_{j}^{\text{IC}}\psi_{i,j}+\sum_{j=1}^{n}\alpha_{j}^{\text{EC}}\theta_{i,j}+\alpha^{\text{CSF}}\omega_{i}$ with $\sum_{j=1}^{n}\left(\alpha_{j}^{\text{IC}}+\alpha_{j}^{\text{EC}}\right)+\alpha^{\text{CSF}}=1$. The *ψ*_*i*,*j*_ models the directional IC compartment diffusion for each fibre bundle using $T_{j}^{\text{IC}}={\chi_{0}}_{j}v_{j}v_{j}^{T}$. The EC compartment with hindered diffusion uses the representation (A6) for *θ*_*i*,*j*_. The isotropic diffusion *ω*_*i*_ uses **T** ^CSF^=*χ*_3_ **I**. This stage keeps the PDDs fixed and only adjusts the *α*'s and *χ*'s of the three compartments.

The parameters of the models were estimated using the training dataset: the *b* values using the equation by Stejskal and Tanner^88^ and the *S*_0_ values as the median of the gradient–free signals with equal echo time per voxel. The initial basis comprises 33 PDDs distributed in the unitary sphere. The bounds *χ*_{0,1}_∈[1,39]×10^−4^ and $\chi_{\left\{2,3\right\}}\in [1,9]\times 10^{-4}{\text{mm}}^{2}/s$ and the LASSO regularization parameter (equals 1.7) was tuned by hand such as to provide the minimum error. The best multi‐tensorial model for both algorithms was used for each voxel to predict the corresponding unseen data.

### A.2 Signal models

#### A.2.1 Alipoor (Chalmers, Sweden)

The diffsuion MRI signal is modelled as a fourth‐order symmetric tensor as proposed by Özarslan and Mareci.^89^ Let **g**_*i*_=[*x*_*i*_ *y*_*i*_ *z*_*i*_] and $a_{i}=[z_{i}^{4}\;4y_{i}z_{i}^{3}\;6y_{i}^{2}z_{i}^{2}\;4y_{i}^{3}z_{i}\;y_{i}^{4}\;4x_{i}z_{i}^{3}\;12x_{i}y_{i}z_{i}^{2}\;12x_{i}y_{i}^{2}z_{i}\;4x_{i}y_{i}^{3}$ $\;6x_{i}^{2}z_{i}^{2}\;12x_{i}^{2}y_{i}z_{i}$ $\;6x_{i}^{2}y_{i}^{2}$ $4x_{i}^{3}z_{i}\;4x_{i}^{3}y_{i}\;x_{i}^{4}\overset{T}{]}$ be a gradient encoding direction and the corresponding design vector, respectively. The diffusion signal is then described by

(A7) $$S(g_{i})=S_{0}\exp\left(\frac{-TE}{T_{2}}\right)\exp(-bt^{T}a_{i})$$

where *S*(**g**_*i*_) is the measured signal when the diffusion sensitizing gradient is applied in the direction **g**_*i*_, *S*_0_ is the observed signal in the absence of such a gradient, *b* is the diffusion‐weighting factor and $t\in R^{15}$ contains the distinct entries of a fourth‐order symmetric tensor. Note that *d*(**g**_*i*_,**t**)=*d*(**g**_*i*_) is used for simplification. Given measurements in *N*>15 different directions, the least‐squares (LS) estimate of the diffusion tensor is $\hat{t}={\left(G^{T}G\right)}^{-1}G^{T}y$, where **G** is an *N*×15 matrix defined by **G**=[**a**_1_ **a**_2_⋯**a**_*N*_]^*T*^ and $y_{i}=-b^{-1}\ln(S(g_{i})/S_{0})$. We use the weighted LS tensor estimation method in Alipoor et al.^90^ to mitigate the influence of outliers.

To estimate the diffusion signal for a given acquisition protocol with *T* *E*=*T* *E*_*x*_, *b*=*b*_*x*_ and *δ*=*δ*_*x*_, the two non‐diffusion‐weighted measurements with the closest *T* *E* to *T* *E*_*x*_ (among measurements with *δ*=*δ*_*x*_) are used to estimate *T*_2_ and *S*_0_ for each voxel. Then, data from the closest shell to *b*_*x*_(among shells with *δ*=*δ*_*x*_) are used to estimate the tensor describing the underlying structure.

#### A.2.2 Sakaie–Tatsuoka–Ghosh (Cleveland, USA): an empirical approach

As the extent of *q* space in the dataset is unusually comprehensive, we chose a simple, generic approach to gain intuition. Visual inspection suggested use of a restricted and hindered component, each with angular variation:

(A8) $$S_{i}=A_{TE_{i}}(fR_{i}+(1-f)\exp(-b_{i}D_{i}))$$

where *S*_*i*_ is the predicted signal for a signal acquired with *T* *E*_*i*_, *b*_*i*_. $A_{TE_{i}}$ is the median signal at a given TE with no diffusion weighting. Fitted parameters are *f*, the volume fraction of *R*_*i*_, the restricted component, and *D*_*i*_, the diffusivity. *R*_*i*_ and *D*_*i*_ are modelled as spherical harmonics with real, antipodal symmetry^91^ with maximum degree 4. The model has 31 fit parameters for each voxel. Data were fitted using using a non‐linear least‐squares algorithm (lsqcurvefit, MATLAB). Prior to the fit, data points with non‐zero bvalue that had a signal higher than the median of the *b*=0 signal plus 1.4826 times the median absolute deviation were excluded. Shells with a normalized median signal smaller than that of shells with lower bvalues were also excluded. Normalization was performed by dividing by the median of the *b*=0 signal with the same TE.

#### A.2.3 Fick (INRIA, France): a spatio‐temporal functional basis to represent the diffusion MRI signal

We use our recently proposed spatio‐temporal (3D+t) functional basis^61^ to represent the diffusion MRI signal simultaneously over three‐dimensional wave vector **q** and diffusion time *τ*. Based on Callaghan's theoretical model of spatio‐temporal diffusion in pores,^92^ our basis represents the 3D+t diffusion signal attenuation *E*(**q**,*τ*) as a product of a spatial and temporal functional basis as

(A9) $$E(q,\tau )=\overset{N_{\max}}{\underset{N=0}{\sum }}\sum_{\left\{jlm\right\}}\overset{O_{\max}}{\underset{o=0}{\sum }}c_{\left\{jlmo\right\}}\;S_{jlm}(q,u_{s})T_{o}(\tau ,u_{t})$$

where *T*_*o*_ is our temporal basis with basis order *o* and *S*_*j**l**m*_ is the spatial isotropic MAP‐MRI basis^29^ with radial and angular basis orders *j*, *l* and *m*. Here, $N_{\max}$ and $O_{\max}$ are the maximum spatial and temporal order of the bases, which can be chosen independently. We formulate the bases themselves as

(A10) $$\begin{array}{ccc} S_{jlm}(q,u_{s}) & =\sqrt{4\text{π}}i^{-l}{\left(2{\text{π}}^{2}u_{s}^{2}q^{2}\right)}^{l/2}{\text{e}}^{-2{\text{π}}^{2}u_{s}^{2}q^{2}} & \\ & \;\times L_{j-1}^{l+1/2}\left(4{\text{π}}^{2}u_{s}^{2}q^{2}\right)Y_{l}^{m}(u) & \\ T_{o}(\tau ,u_{t}) & =\exp(-u_{t}\tau /2)L_{o}(u_{t}\tau ) & \end{array}$$

with *u*_*s*_ and *u*_*t*_ the spatial and temporal scaling functions, $Y_{l}^{m}$ the spherical harmonics and *L*_*o*_ a Laguerre polynomial. We calculate the spatial scaling *u*_*s*_ by fitting an isotropic tensor to the TE‐normalized signal attenuation *E*(**q**,·) for all **q**. Similarly, we compute *u*_*t*_ by fitting an exponential $e^{-u_{t}\tau /2}$ to *E*(·,*τ*) for all *τ*. We fit our basis using Laplacian‐regularized least squares in the following steps. We first denote Ξ _*i*_(**q**,*τ*,*u*_*s*_,*u*_*t*_)=*S*_*j**l**m*(*i*)_(**q**,*u*_*s*_)*T*_*o*(*i*)_(*τ*,*u*_*t*_) with *i*∈{1…*N*_coef_}, with *N*_coef_ the number of fitted coefficients. We then construct a design matrix $Q\in R^{N_{\text{data}\;}\times N_{\text{coef}\;}}$ with $Q_{ik}=S_{N_{i}}(A,q_{k})T_{o_{i}}(\tau_{k},u_{t})$. The signal is then fitted as **c**=argmin_**c**_∥**y**−**Qc**∥^2^+*λ* *U*(**c**) with **y** the measured signal, **c** the fitted coefficients and *λ* the weight for our analytic Laplacian regularization *U*(**c**). We used generalized cross‐validation^93^ to find the optimal regularization weighting *λ* in every voxel. In our submitted results, we used a spatial order of 8 and a temporal order of 4, resulting in 475 fitted coefficients.

#### A.2.4 Rivera (CIMAT, Mexico): baseline method: robust regression

We regard this very simplistic model as a baseline for other model‐based methods. It assumes as little information as possible from the diffusion signal. The vector of independent variables is *x*_*i*_=[*g*_*i*_,|*G*|_*i*_,Δ_*i*_,*δ*_*i*_,*T* *E*_*i*_,*b*_*i*_], containing the gradient strength *g*, the echo time *T* *E* and the *b* value *b*. Given signal *s*_*i*_, we then estimate the parameters of the linear regression model:

(A11) s=Xθ+ϵ

where *θ*∈*R* ^23^ is the unknown vector of coefficients, *ϵ* is the residual error and

$$X=\left[x,x{|}^{2},\Delta \;\delta ,\Delta \;TE,\Delta \;b,\delta \;TE,\delta \;b,TE\;b,1\right]$$

is the *matrix design* (*x*|^2^ is obtained from squaring each element of the matrix x). To account for outliers, we estimate *θ* with a weighted (robust) least‐squares approach using the Lasso regularization:

(A12) $$\theta^{t+1}=\underset{\theta }{arg min}\;∥W^{t}(X\theta -y){∥}_{2}^{2}+\lambda ∥\theta {∥}_{1}$$

where *W* ^0^ is the identity matrix and each subsequent *W* is computed via

(A13) $$W^{t+1}=\text{diag}(v_{i}^{t+1}w_{i}^{t+1})$$

with outlier weighting in $\omega_{i}^{t+1}=\kappa^{2}/(\kappa^{2}+{\left(y_{i}-X\theta_{i}^{t+1}\right)}^{2})$ through *κ*, an arbitrary parameter that controls the outlier sensitivity. The protocol weight

(A14) $$\begin{array}{ccc} v_{i}^{t+1} & ={\text{mean}}_{j\in \Omega_{i}}\{w_{j}^{t+1}\} & \\ \text{and} & \\ \Omega_{i} & =\{j:TE_{j}=TE_{i},|G_{j}|=|G_{i}|\} \end{array}$$

computes a confidence factor for the complete protocol.

Equations A12 and A13 are iterated three times. The final estimated signal is computed using (A11), using the protocol of the unseen signal.
